# Rapamycin improves Graves’ orbitopathy by suppressing CD4^+^ cytotoxic T lymphocytes

**DOI:** 10.1172/jci.insight.160377

**Published:** 2023-02-08

**Authors:** Meng Zhang, Kelvin K.L. Chong, Zi-yi Chen, Hui Guo, Yu-feng Liu, Yong-yong Kang, Yang-jun Li, Ting-ting Shi, Kenneth K.H. Lai, Ming-qian He, Kai Ye, George J. Kahaly, Bing-yin Shi, Yue Wang

**Affiliations:** 1 Department of Endocrinology, The First Affiliated Hospital of Xi’an Jiaotong University, Xi’an, China.; 2 Department of Ophthalmology and Visual Sciences, The Chinese University of Hong Kong, Hong Kong, China.; 3 Department of Ophthalmology and Visual Science, The Prince of Wales Hospital, Hong Kong, China.; 4 Biobank of The First Affiliated Hospital of Xi’an Jiaotong University, Xi’an, China.; 5 Genome Institute and; 6 Center for Mathematical Medical, The First Affiliated Hospital of Xi’an Jiaotong University, Xi’an, China.; 7 Department of Ophthalmology, Tangdu Hospital, Air Force Military Medical University, Xi’an, China.; 8 Department of Endocrinology, Beijing Tongren Hospital, Capital Medical University, Beijing, China.; 9 Department of Ophthalmology, Tung Wah Eastern Hospital, Hong Kong, China.; 10 MOE Key Laboratory for Intelligent Networks & Network Security and; 11 School of Automation Science and Engineering, Faculty of Electronic and Information Engineering, Xi’an Jiaotong University, Xi’an, China.; 12 School of Life Science and Technology, Xi’an Jiaotong University, Xi’an, China.; 13 Faculty of Science, Leiden University, Leiden, Netherlands.; 14 Molecular Thyroid Lab, Department of Medicine I, Johannes Gutenberg University Medical Center, Mainz, Germany.

**Keywords:** Autoimmunity, Endocrinology, Autoimmune diseases, Immunotherapy, T cells

## Abstract

CD4^+^ cytotoxic T lymphocytes (CTLs) were recently implicated in immune-mediated inflammation and fibrosis progression of Graves’ orbitopathy (GO). However, little is known about therapeutic targeting of CD4^+^ CTLs. Herein, we studied the effect of rapamycin, an approved mTOR complex 1 (mTORC1) inhibitor, in a GO mouse model, in vitro, and in patients with refractory GO. In the adenovirus-induced model, rapamycin significantly decreased the incidence of GO. This was accompanied by the reduction of both CD4^+^ CTLs and the reduction of orbital inflammation, adipogenesis, and fibrosis. CD4^+^ CTLs from patients with active GO showed upregulation of the mTOR pathway, while rapamycin decreased their proportions and cytotoxic function. Low-dose rapamycin treatment substantially improved diplopia and the clinical activity score in steroid-refractory patients with GO. Single-cell RNA-Seq revealed that eye motility improvement was closely related to suppression of inflammation and chemotaxis in CD4^+^ CTLs. In conclusion, rapamycin is a promising treatment for CD4^+^ CTL-mediated inflammation and fibrosis in GO.

## Graphical abstract

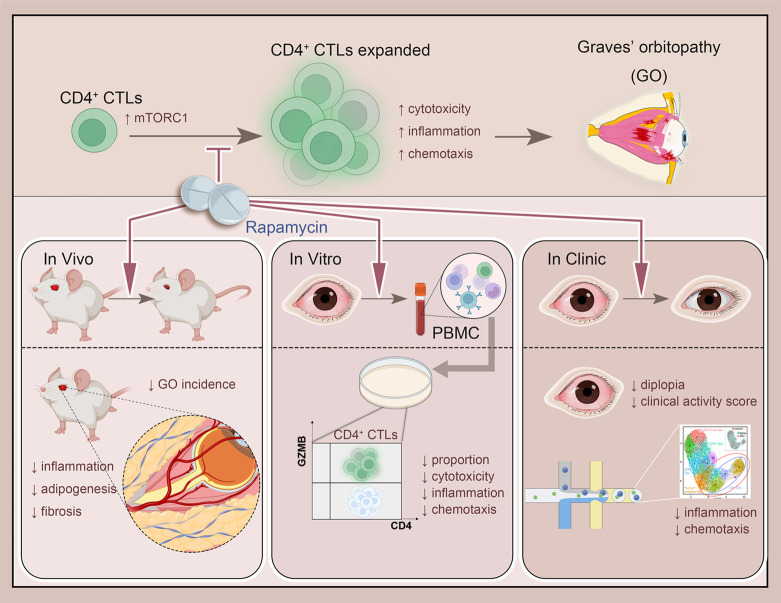

## Introduction

Graves’ disease (GD), an autoimmune disorder, is the main cause of hyperthyroidism in adults (1). Graves’ orbitopathy (GO) is the most common extrathyroidal manifestation of GD, with an incidence up to 50% (2). GO results in visual disability and cosmetic disfiguration, which significantly decrease patients’ quality of life (2, 3). The pathological changes in GO are orbital inflammation, adipogenesis, and fibrosis mediated largely by CD4^+^ T cells (4). Glucocorticoids have been the mainstay GO treatment since the last century, and the effect of steroid monotherapy remains suboptimal (3). Recently, mycophenolate, rituximab (anti-CD20), and tocilizumab (anti–IL-6) were reported to achieve some benefits in clinical trials (5–7). Teprotumumab, which targets the insulin-like growth factor 1 receptor pathway in orbital fibroblasts, was also found to be effective at improving exophthalmos and diplopia (8). Thus, it is imperative to explore the underlying molecular and pathogenic mechanisms for potentially novel therapeutic targets to improve the outcome of patients with GO.

CD4^+^ T cells have been reported to initiate and perpetuate orbital inflammation in GO (9). Recently, we reported what we believe to be a novel proinflammatory CD4^+^ cytotoxic T lymphocyte (CTL) subtype that drives the progression of GO in patients with GD (10). CD4^+^ CTLs marked by granzyme B (GZMB) and IFN-γ (IFNG) may migrate from the circulation to orbits and trigger orbital inflammation and tissue remodeling. Genes involved in inflammation and cytotoxicity were also significantly upregulated in GO (11). Interestingly, CD4^+^ CTLs with an effector/memory phenotype were expanded in inflamed tissue sites and secreted the profibrotic cytokine IFNG in IgG4-related disease (12, 13). They were also identified as drivers of lung inflammation and fibrosis in severe coronavirus disease 2019 (COVID-19) (14). Thus, we propose that CD4^+^ CTLs are a potentially novel target for inflammation, cytotoxicity, and fibrosis in GO.

Rapamycin is a mechanistic/mammalian inhibitor that mainly targets mTOR complex 1 (mTORC1) (15). Activation of mTORC1-dependent pathways is required for the differentiation and functions of effector CD8^+^ CTLs (16, 17). Enhanced mTORC1 signaling in CTLs results in an increase in terminally differentiated GZMB and perforin-expressing effector cells (16). It also leads to increased IFNG and TNF-α (TNFA) production and cellular proliferation (16). Proteomic studies have confirmed the role of mTORC1 in selective expression of effector molecules, including granzymes, perforin, TNF, and IFNG in CTLs (17). Moreover, chemotaxis by activated CD4^+^ T cells mediated by CCL5 is also dependent on mTOR (18). Thus, we hypothesized that rapamycin could be an effective intervention targeting CD4^+^ CTLs and a potential preventive and therapeutic agent in GO.

Here, we investigated the role of mTORC1 in regulating GO-specific CD4^+^ CTLs and evaluated the potential of rapamycin as an immunotherapy via a GO mouse model, in vitro, and clinical intervention studies.

## Results

### Rapamycin decreases CD4^+^ CTLs’ accumulation and suppresses their inflammatory, chemotactic, and cytotoxic functions in GO mice.

**Figure 1:**
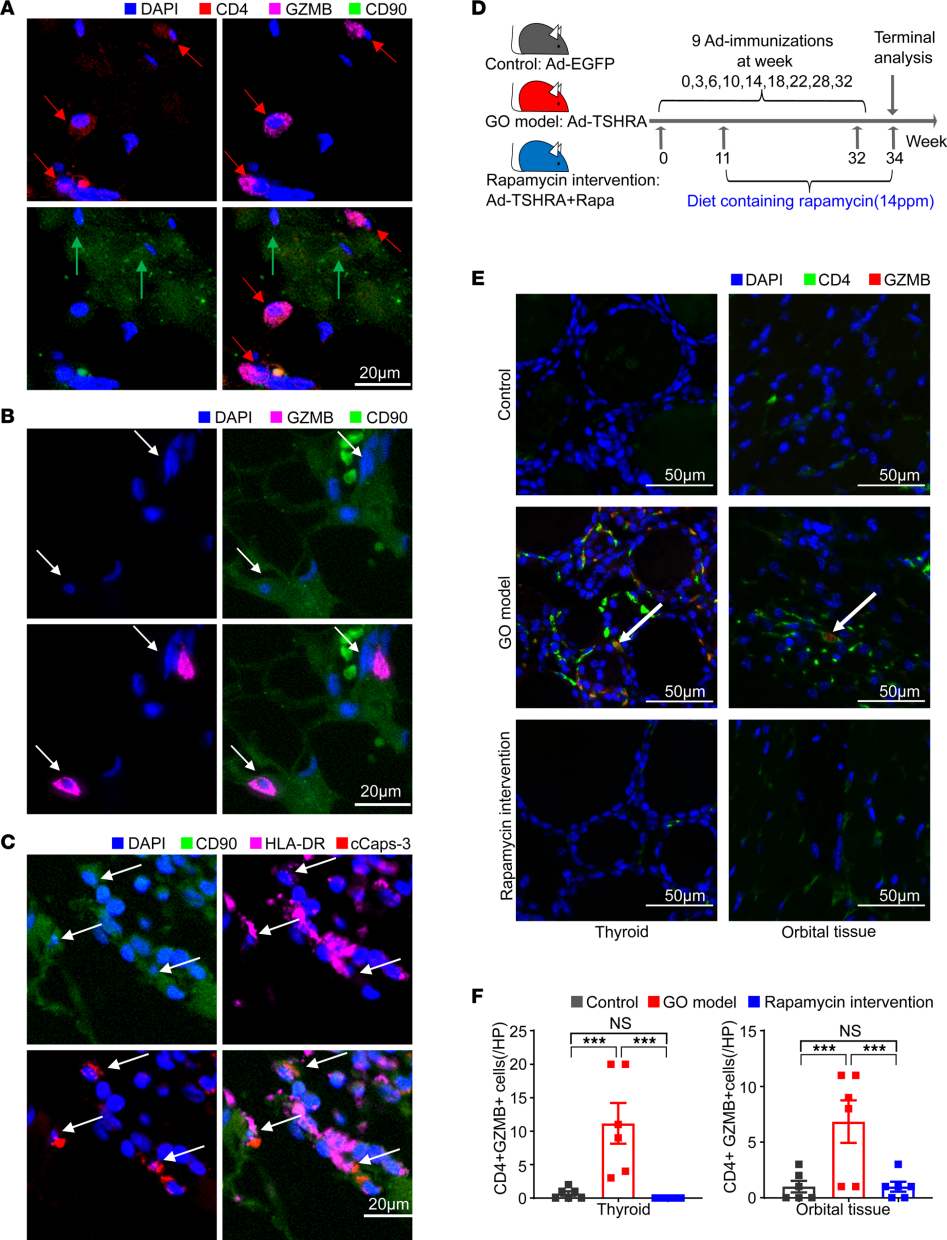
Rapamycin significantly decreases CD4^+^ CTL accumulation and suppresses their function in GO mice. (**A**) Representative multicolor immunofluorescence image showing CD4^+^GZMB^+^ T cells (indicated by red arrows) accumulate in the vicinity of CD90^+^ fibroblasts (indicated by green arrows) in orbital tissue from patients with GO. (**B**) Represe ntative multicolor immunofluorescence image showing the CD90^+^ fibroblasts with GZMB visible within the cytosol, suggesting that fibroblasts may represent targets of CD4^+^ CTL–directed cytotoxicity. (**C**) Multicolor immunofluorescence images of cCasp-3^+^ and HLA-DR^+^CD90^+^ fibroblasts (indicated by arrows) in orbital tissue from patients with GO. (**D**) Animal study design. Control mice were immunized with Ad-EGFP, GO mice were immunized with Ad-TSHRA, and rapamycin intervention mice were immunized with Ad-TSHRA while fed a diet containing rapamycin from week 11 until the end of the experiment (14 ppm, 23 weeks). (**E**) Representative example of thyroid and orbital tissue from the 3 groups of mice by fluorescent multiplex IHC showing coexpression of CD4 and GZMB (indicated by white arrows). (**F**) Bar plots exhibited counts of CD4^+^GZMB^+^ T cells in thyroid and orbital tissue from the 3 groups of mice (*n* = 6 in each group). Values represent the mean ± SEM. ***P < 0.001, by 2-tailed, unpaired Mann-Whitney-Wilcoxon rank test for **F**.

Recently, we have reported GO-specific CD4^+^ CTLs that expanded in PBMCs and accumulated in orbital tissues (10). In this study, CD4^+^GZMB^+^ accumulated in the vicinity of CD90^+^ fibroblasts were observed again in the orbital tissue of patients with GO, as well as the cleaved caspase-3 (cCasp-3) accumulated in HLA-DR–expressing CD90^+^ fibroblasts (Figure 1, A–C).

To investigate the effect of rapamycin on the accumulated CD4^+^ CTLs, a GO mouse model was established as previously reported (11). The expression of genes related to the mTOR pathway was significantly increased in GO mice compared with control (*P* < 0.05; Supplemental Figure 1A; supplemental material available online with this article; https://doi.org/10.1172/jci.insight.160377DS1). In this study, the mice were divided into control, GO model, and rapamycin treatment groups (Figure 1D; for further details, see Methods). Compared with the GO group, rapamycin treatment markedly decreased the CD4^+^GZMB^+^ cells in both orbital tissues (3.17 cells/high-power field [HP] vs. 0.33 cells/HP, *P* < 0.001) as well as in the thyroid glands (10.78 cells/HP vs. 0 cells/HP, *P* < 0.001; Figure 1, E and F).

In addition, gene expressions in splenocytes were analyzed. The expression of genes related to the immune response, cell-cell adhesion, inflammation, chemotaxis, cytolysis, and cell killing were significantly increased in GO mice compared with control (*P* < 0.05). No significant difference was detected between rapamycin-treated mice and control (Supplemental Figure 1B and Supplemental Table 1). Gene set enrichment analysis (GSEA) showed that compared with GO mice, rapamycin-treated mice showed significant downregulation of pathways related to adhesion, inflammation, and cell killing (Supplemental Figure 1C).

### Rapamycin effectively ameliorates orbitopathy and hyperthyroidism in GO mice.

**Figure 2:**
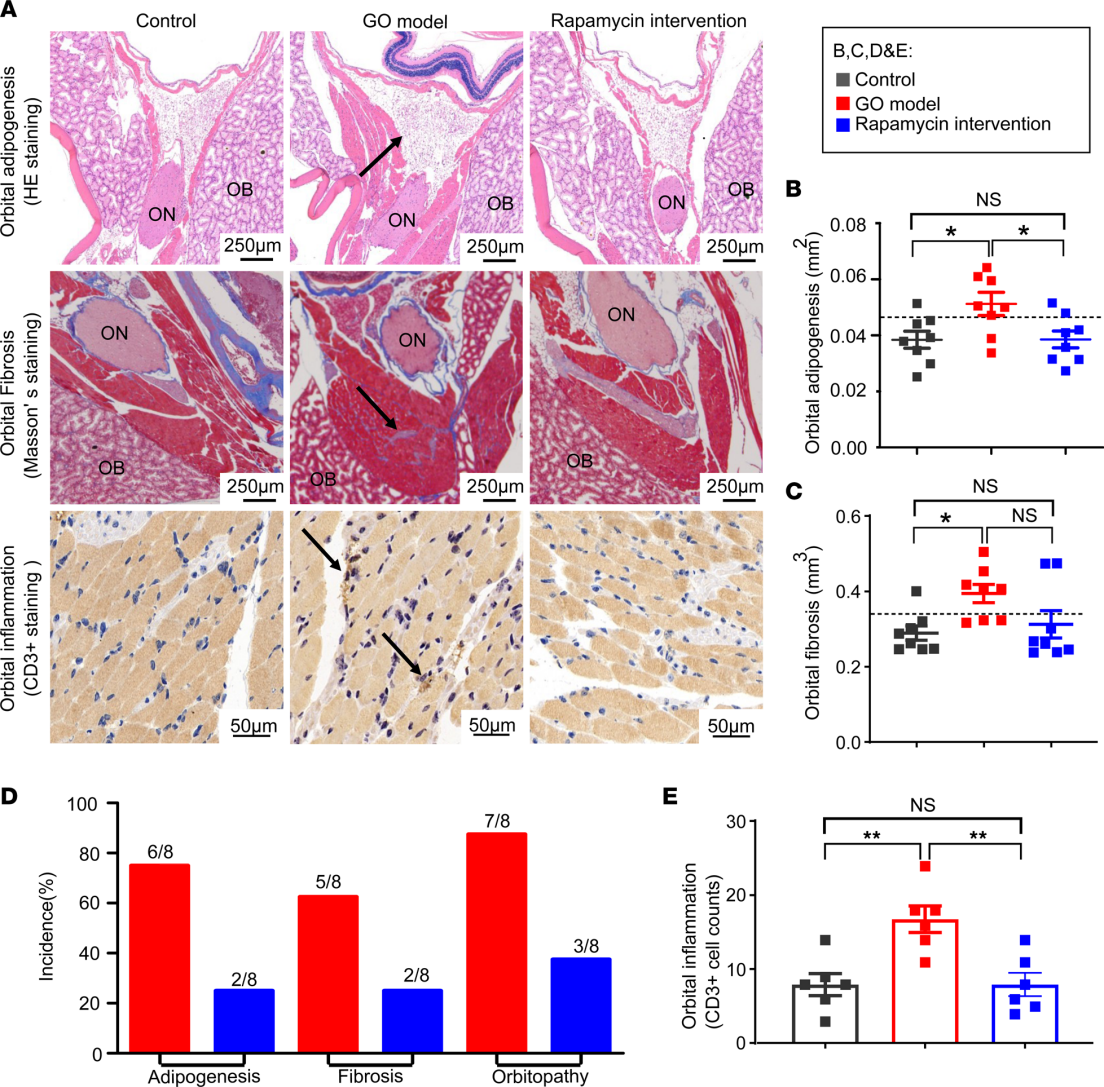
Rapamycin significantly ameliorates orbitopathy in GO mice. (**A**) Representative microscopy images of orbital fibrosis, adipogenesis, and inflammation in mice (indicated by black arrows). ON, optic nerve; OB, orbital bones. (**B** and **C**) Scatterplots of fibrosis volume and adipogenesis area in orbits from the 3 groups of mice (*n* = 8 for each group). The dashed line shows the mean + 2 SDs of the value for control mice, which was considered the normal range. (**D**) The incidences of fibrosis, adipogenesis, and orbitopathy in GO mice and rapamycin intervention mice. The incidences of fibrosis and adipogenesis: the mean + 2 SDs of control mice was regarded as the normal range, and higher values indicated significant positivity. The incidence of orbitopathy: existing orbital pathogenesis including fibrosis and adipogenesis. (**E**) Bar plots exhibited counts of CD3^+^ T cells in orbital tissue from the 3 groups of mice (*n* = 6 for each group). Values represent the mean ± SEM. *P < 0.05, **P < 0.01, and NS *P* > 0.05, by 1-way ANOVA and post-ANOVA, pairwise, 2-group comparisons with Tukey’s method for **B**, **C**, and **E**.

To evaluate the effects of rapamycin on retrobulbar adipogenesis and fibrosis in GO, orbital sections were stained with Masson’s trichrome and H&E. Compared with GO mice, rapamycin-treated mice showed a decrease in retrobulbar adipogenesis (*P* = 0.041; Figure 2, A and B). Taking the mean + 2 SDs of the control group as the normal range, rapamycin treatment decreased the incidence of orbitopathy from 87.5% to 37.5% and reduced the incidences of adipogenesis (from 75% to 25%) and fibrosis (from 62.5% to 25%) (Figure 2, B–D; and Supplemental Table 2). Compared with GO mice, rapamycin treatment also significantly decreased orbital inflammation, as indicated by evaluation of CD3^+^ cells in orbital tissue (*P* = 0.004; Figure 2, A and E) (19).

**Figure 3:**
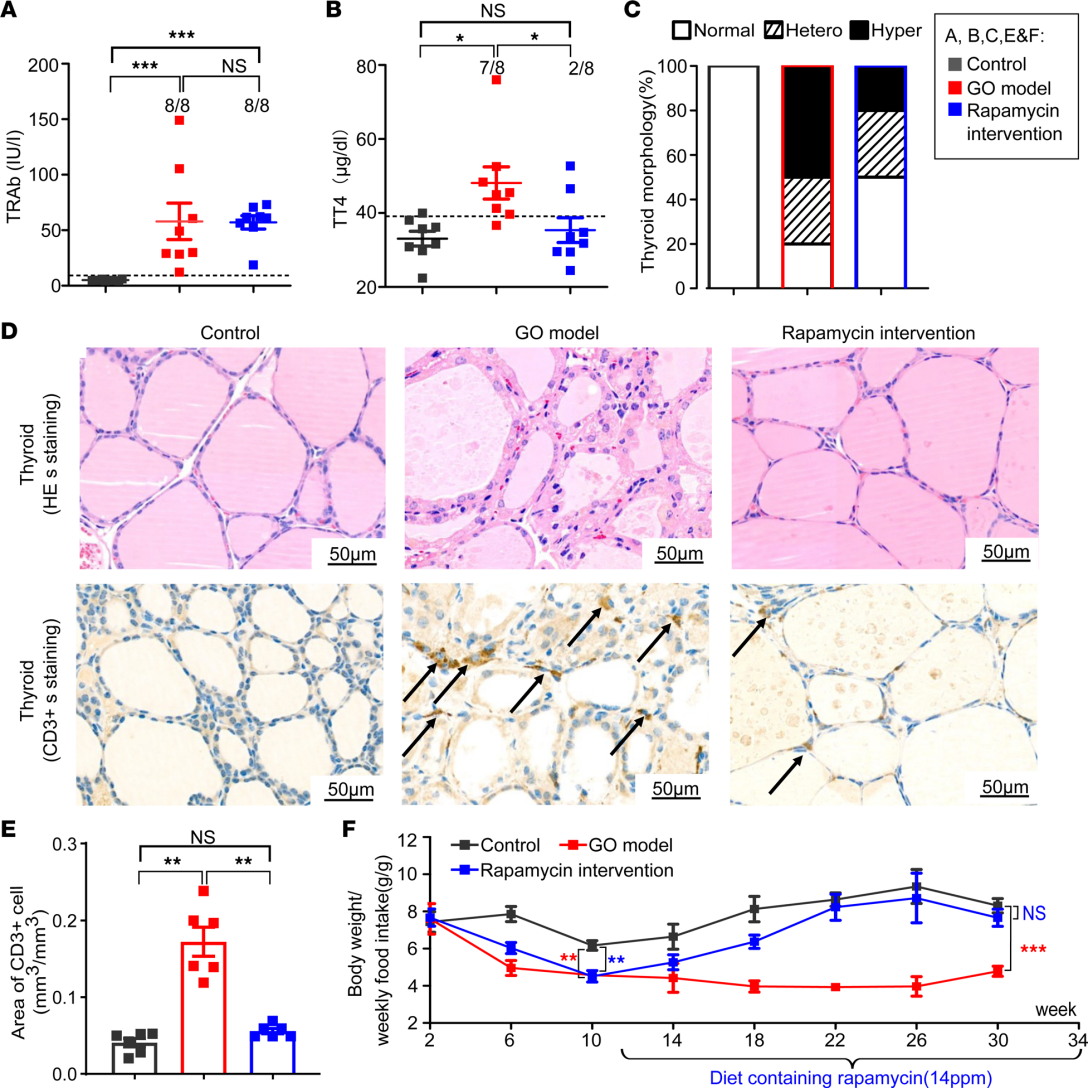
Rapamycin significantly ameliorates hyperthyroidism in GO mice. (**A** and **B**) Scatterplots of TRAb and TT4 levels of the 3 groups of mice (*n* = 8 for each group). The dashed line shows the mean + 2 SDs of the value for control mice, which was regarded as the normal range. (**C**) Percentage bar plots which indicated the distribution of thyroid morphologic types in the 3 groups of mice (*n* = 8 for each group). The thyroid morphology was divided into 3 types: normal, hyperplastic (hyper), and heterogeneous (hetero). The typical hyperplastic changes: hyperplasia of follicular cells, which were cuboidal or tall columnar cells and even led to papillary folds and protrusions in the follicular cavity. Some of the glands showed a heterogeneous morphology with a mixture of normal and hyperplastic areas. (**D**) Representative microscopy images for morphology of thyroid in the 3 groups of mice. The morphology of GO model mice exhibited typical hyperplastic changes. CD3^+^ cells in the thyroid are indicated by black arrows, indicating inflammation in the thyroid. (**E**) Bar plots exhibited infiltrated area of CD3^+^ T cells in the thyroid from the 3 groups of mice (*n* = 6 for each group). (**F**) The ratio of BW to weekly food intake of mice in the 3 groups during the experiment. The statistical differences between the GO model with control group and the rapamycin intervention group with control group were labeled at the 10th and 30th week, respectively. Values represent the mean ± SEM. *P < 0.05, **P < 0.01, ***P < 0.001, and NS *P* > 0.05, by 2-tailed, unpaired Mann-Whitney-Wilcoxon rank test for **A** and **E** and 1-way ANOVA and post-ANOVA, pairwise, 2-group comparisons with Tukey’s method for **B** and **F**.

The effect of rapamycin treatment on hyperthyroidism was evaluated by thyroid autoantibodies, function, and pathology. There was no difference in serum binding anti–thyrotropin receptor (anti-TSHR) autoantibody (TRAb) levels between the GO and rapamycin treatment groups (Figure 3A). Taking the mean + 2 SDs of total thyroxine (TT4) values in the control group as the normal range, rapamycin treatment decreased the frequency of hyperthyroidism from 87.5% to 25% (*P* = 0.036; Figure 3B). The results were confirmed by the pathology of the thyroid glands in rapamycin-treated mice, which showed a decrease in the hyperplastic change (50% vs. 20%; Figure 3, C and D). The infiltration of CD3^+^ T cells in the thyroids was also decreased in rapamycin-treated mice (*P* = 0.002; Figure 3, D and E). Additionally, the ratio of BW to weekly food intake in GO mice gradually increased to normal after rapamycin treatment (Figure 3F).

### Rapamycin decreases the frequency of CD4^+^ CTLs in PBMCs of patients with GO by targeting mTORC1.

**Figure 4:**
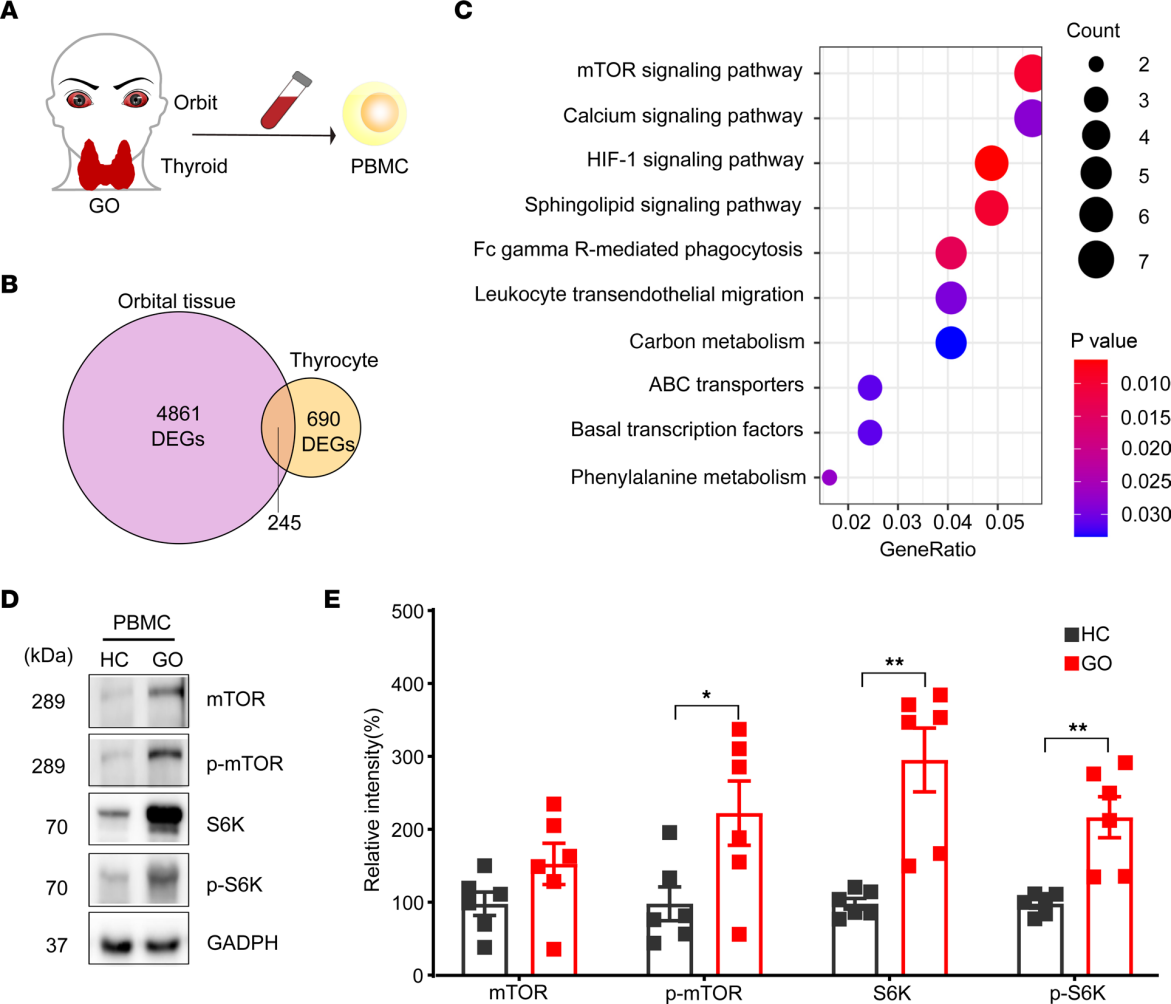
The mTORC1 signaling pathway is upregulated in the orbital tissue, thyrocytes, and PBMCs of patients with GO. (**A**) Orbits, thyroids, and PBMCs from patients with GO were all investigated to determine the key pathway involved in GO pathogenesis. (**B**) A Venn diagram of differentially expressed genes (DEGs) in the GO gene set identified in the GSE58331 data set (orbital tissues; GO vs. HC) and in the GSE9340 data set (thyrocytes; GO vs. GD). A total of 245 GO-specific genes were identified. (**C**) Top 10 enrichment signatures revealed by KEGG analysis of the 245 GO-specific genes. The mTOR signaling pathway was the top enriched pathway in GO. *P* values are indicated by color. The number of DEGs in enrichment signatures were indicated by circle size. (**D**) Representative images of Western blot, which analyzed the key signaling molecules involved in mTORC1 signaling pathways from PBMCs of HCs and patients with GO. (**E**) Bar plots for the relative intensities in the Western blot of mTOR, p-mTOR, S6K, and p-S6K in the PBMCs of healthy controls and patients with GO (*n* = 6 for each group). Values represent the mean ± SEM. *P < 0.05 and **P < 0.01, by 2-tailed, unpaired Mann-Whitney-Wilcoxon rank test and independent-sample 2-tailed *t* tests for **E**.

The orbit, the thyroid gland, and the PBMCs were all investigated to confirm the key pathogenic pathways in patients with GO (Figure 4A). An analysis was conducted of 2 microarray data sets for orbital tissues and thyrocytes of patients with GO (Supplemental Figure 2) (20). Kyoto Encyclopedia of Genes and Genomes (KEGG) analysis showed that mTOR signaling was the top enriched pathway in GO (Figure 4, B and C). PBMCs from patients with GO showed significantly increased levels of phosphorylated mTOR (p-mTOR), downstream signaling molecule ribosomal protein S6 kinase (70 kDa) (S6K), and its phosphorylated state p-S6K (*P* = 0.032, 0.002, and 0.002, respectively; Figure 4, D and E).

**Figure 5:**
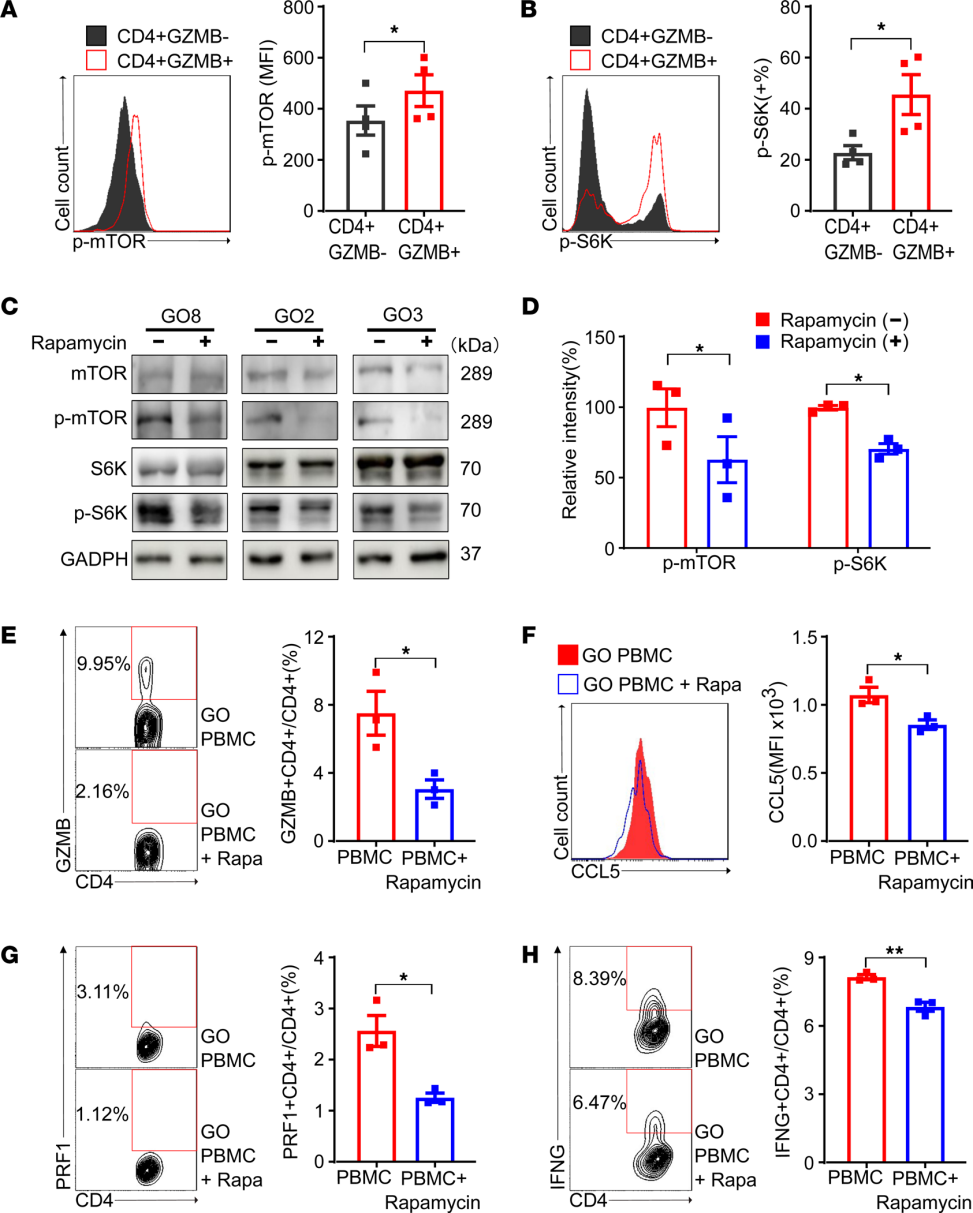
The inhibition of mTORC1 by rapamycin decreases the frequency and functions of CD4^+^ CTLs in PBMCs of patients with GO. (**A** and **B**) Flow cytometric analysis of key signaling molecules in mTORC1 signaling pathways in CD4^+^GZMB^+^ cells and CD4^+^GZMB^–^ cells from patients with GO (*n* = 4). FACS plots and bar plots were presented for intracellular p-mTOR (**A**) and its downstream molecule p-S6K (**B**). CD4^+^GZMB^+^ cells expressed higher levels of p-mTOR and p-S6K than CD4^+^GZMB^–^ cells. (**C**) Representative images of Western blot, which analyzed the key signaling molecules involved in mTORC1 signaling pathways from PBMCs treated with or without rapamycin. The PBMCs were obtained from patients with GO and incubated with or without rapamycin (100 nM, 48 hours). (**D**) Bar plots for relative intensities in the Western blot of p-mTOR and p-S6K in PBMCs treated with or without rapamycin (*n* = 3). (**E**) Flow cytometric analysis of the proportions of CD4^+^ CTLs expressing GZMB in PBMCs treated with or without rapamycin (*n* = 3). FACS plots and bar plots are presented. (**F**) Flow cytometric analysis of CCL5 in CD4^+^GZMB^+^ cells treated with or without rapamycin (*n* = 3). FACS plots and bar plots are presented. (**G** and **H**) Flow cytometric analysis of the proportions of CD4^+^PRF1^+^ and CD4^+^IFNG^+^ cells in PBMCs treated with or without rapamycin (*n* = 3). FACS plots and bar plots are presented. Values represent the mean ± SEM. *P < 0.05 and **P < 0.01, by 2-tailed, paired samples *t* tests for **A** and **B** and 2-tailed, independent samples *t* tests for **D**–**H**;

As CD4^+^ CTLs were shown to be involved in the pathogenesis of GO (Supplemental Figure 3), flow cytometry was performed to investigate the mTORC1. Compared with CD4^+^GZMB^–^ cells, CD4^+^GZMB^+^ cells expressed significantly higher levels of p-mTOR and its downstream molecule p-S6K (*P* = 0.036 and 0.047, respectively; Figure 5, A and B). Incubation of PBMCs from patients with GO with rapamycin promoted the dephosphorylation of mTOR and S6K (*P* = 0.044 and 0.031, respectively; Figure 5, C and D).

Compared with untreated PBMCs, rapamycin-treated PBMCs exhibited significant decreases in the frequency of CD4^+^GZMB^+^ cells (7.55% vs. 3.09%, *P* = 0.033; Figure 5E) and the MFI of CCL5 in CD4^+^GZMB^+^ cells (1,078 vs. 860, *P* = 0.029; Figure 5F), the latter of which is a key chemotactic factor. The proportions of CD4^+^perforin 1^+^ (PRF1^+^) and CD4^+^IFNG^+^ cells also decreased after rapamycin treatment (*P* = 0.014 and 0.005; Figure 5, G and H), suggesting suppressed cytotoxicity and proinflammatory functions, respectively. Rapamycin-treated PBMCs exhibited no differences on the frequency and functions of Tregs, which may be due to the insufficient time and lack of added Treg inducers (TGFB, IL-2, etc.) (21, 22) (Supplemental Figure 4).

### Rapamycin ameliorates diplopia and orbital inflammation in patients with intractable GO.

**Figure 6:**
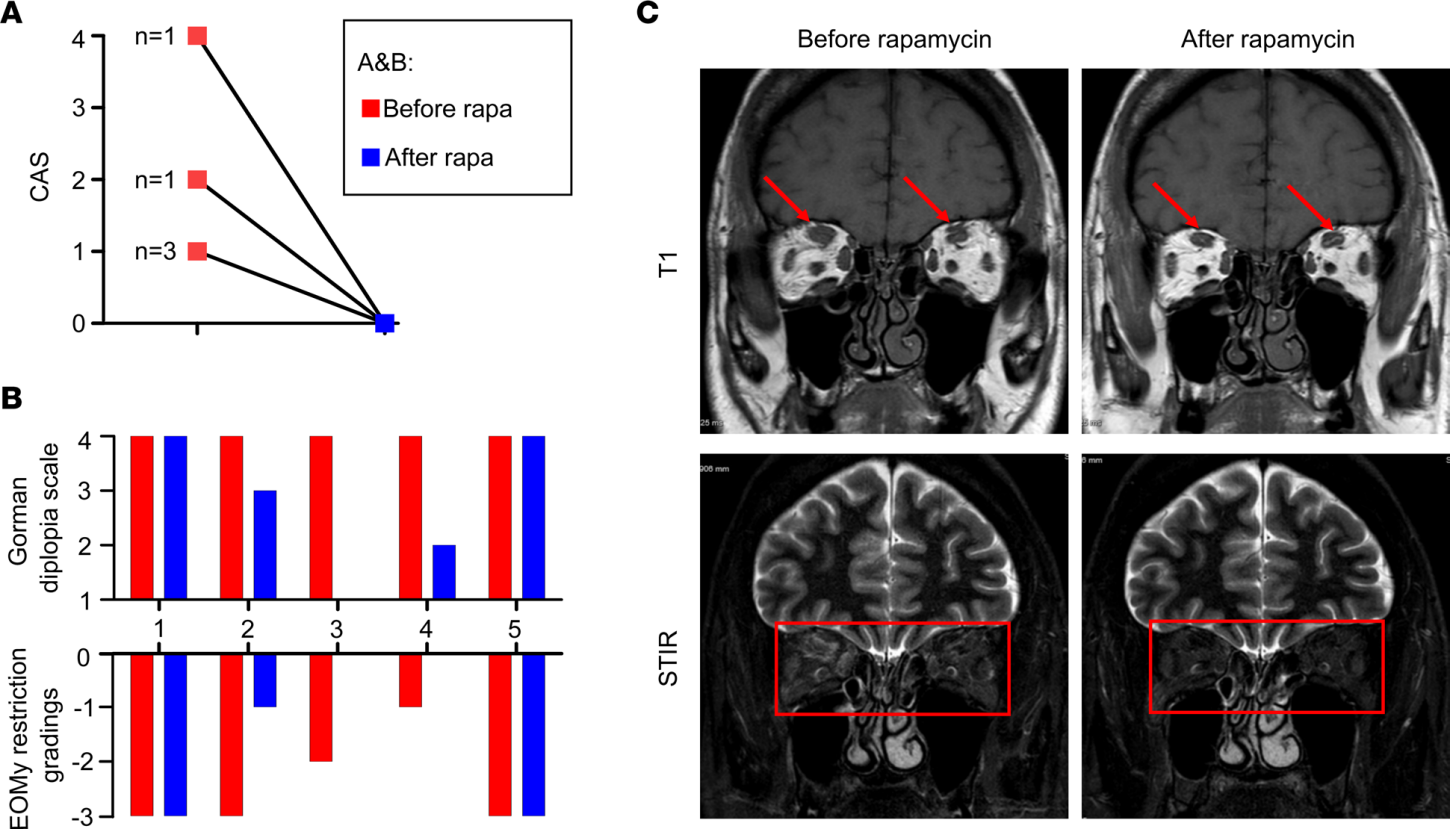
Rapamycin improves orbitopathy and diplopia in patients with refractory GO. (**A**) CAS before and after rapamycin treatment (*n* = 5). (**B**) EOMy restriction gradings and Gorman diplopia scale of individual patients with GO. (**C**) Representative MRI images on T1 weight and short tau inversion recovery (STIR) sequence of good responders before and after rapamycin treatment.

A low-dose rapamycin treatment (2 mg/d) was given during 12 months to patients with steroid-refractory GO with intractable diplopia (*n* = 5; Supplemental Table 3). All 5 patients showed further improvement in the clinical activity score (CAS), with 3 also improving their extraocular muscle motility (EOMy) restriction grading and the related Gorman diplopia scores (Figure 6, A and B). Orbital images of patient 4 showed a decrease of extraocular muscle thickness and edema, consistent with clinical improvement (Figure 6C).

### Single-cell sequencing of CD4^+^ T cells verifies rapamycin suppresses mTORC1 pathway and cytotoxic and inflammatory functions of CD4^+^ CTLs in patients with GO.

**Figure 7:**
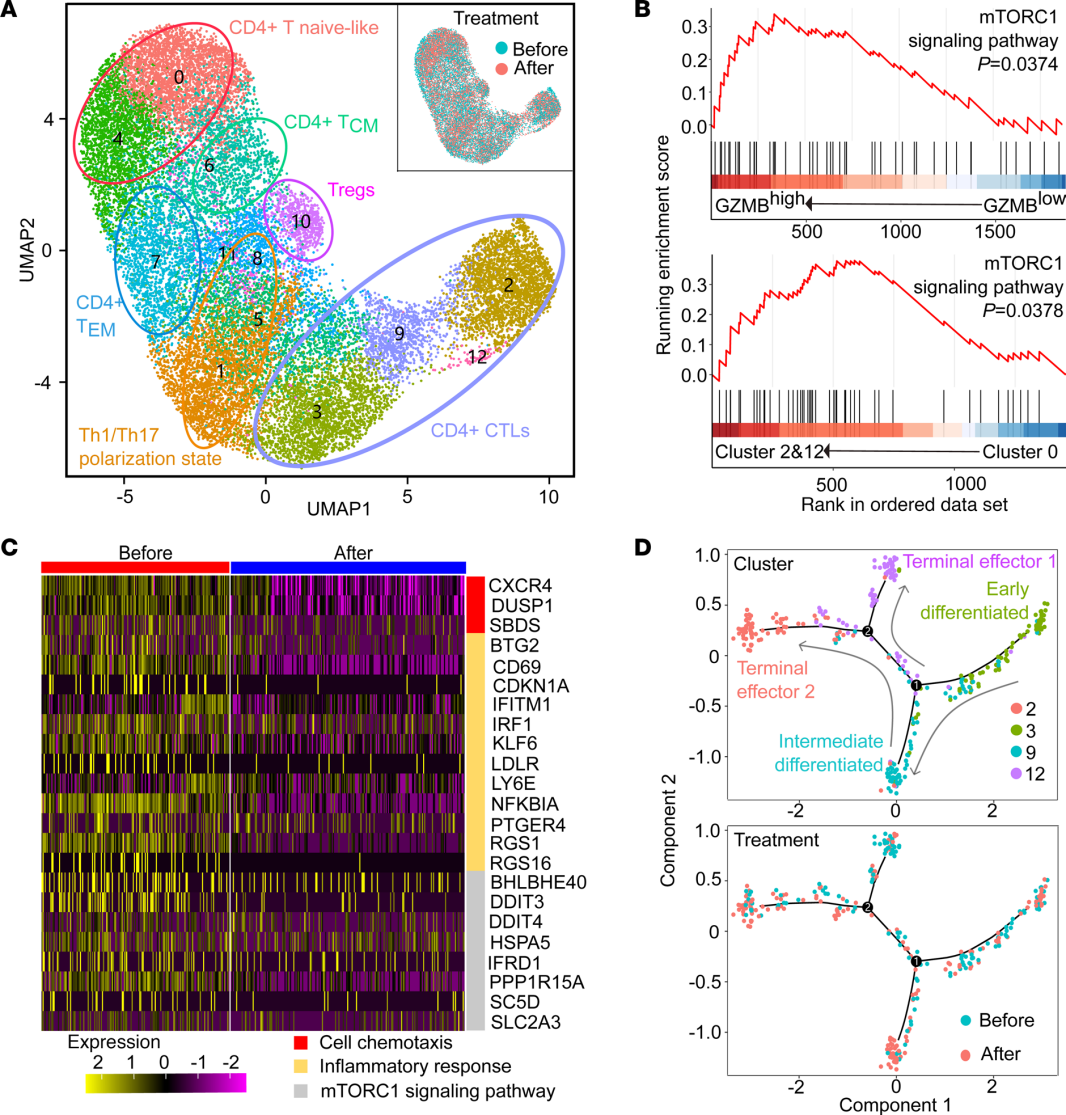
Single-cell sequencing of CD4^+^ T cells demonstrates rapamycin suppresses mTORC1 pathway and cytotoxic and inflammatory functions of CD4^+^ CTLs in patients with GO. (**A**) The graph-based clustering and uniform manifold approximation and projection (UMAP) algorithm were applied in 23,317 CD4^+^ T cells from 2 patients before and after rapamycin treatment. Clusters denoted by the same color scheme were labeled with inferred cell types: CD4^+^ T naive-like cells (CCR7^+^SELL^+^), CD4^+^ T_CM_ cells (SELL^+^CD27^+^), CD4^+^ T_EM_ cells (LIMS1^+^GPR183^+^), CD4^+^ T_regs_ (FOXP3^+^IKZF2^+^), CD4^+^ Th1/Th17-polarized cells (CCR6^+^CXCR3^+^), and CD4^+^ CTLs (GZMA^+^CCL5^+^). The clusters (upper right) denoted by blue and red were labeled with treatment status (before or after rapamycin treatment). (**B**) GSEA enrichment plots for mTORC1 signaling gene set in the transcriptome of CD4^+^GZMB^hi^ versus CD4^+^GZMB^lo^ cells (upper), and in the transcriptome of clusters 2 and 12 versus cluster 0 (bottom), respectively. (**C**) Heatmap of gene expression related to cell chemotaxis, the inflammatory responses, and the mTORC1 signaling pathway in CD4^+^ CTLs before and after rapamycin treatment. The expression was indicated by color. (**D**) Trajectory analysis of CD4^+^ CTLs. Cells on the trajectories are aligned in the order of differentiation (the arrow shape) representing the gradual transition from initial state to cell fate state. The upper trajectory shows the cells colored by cluster. The trajectory below shows the cells colored by treatment status (before or after rapamycin treatment).

To explore the treatment mechanism of rapamycin, we performed single-cell sequencing of CD4^+^ T cells from 2 patients with steroid-refractory GO before and after rapamycin treatment (Figure 7A). Cell clusters were identified based on the expression of signature genes and canonical lineage markers including *CCR7*, selectin L (*SELL*), *CD27*, LIM zinc finger domain containing 1 (*LIMS1*), G protein–coupled receptor 183 (*GPR183*), Forkhead box P3 (*FOXP3*), *CCR6*, IKAROS family zinc finger protein 2 (*IKZF2*), *CXCR3*, *GZMA*, and *CCL5*. A total of 6 cell clusters were identified: CD4^+^CCR7^+^SELL^+^ T naive-like cells; CD4^+^SELL^+^CD27^+^ T central memory (T_CM_) cells; CD4^+^LIMS1^+^GPR183^+^ T effector memory (T_EM_) cells; CD4^+^FOXP3^+^IKZF2^+^ (T_regs_); CD4^+^CCR6^+^CXCR3^+^ TTh1/Th17-polarized cells; and CD4^+^GZMA^+^CCL5^+^ CTLs (Supplemental Figure 5). The GSEA plots of the pretreatment CD4^+^ cells showed upregulation of the mTORC1 signaling pathway in CD4^+^GZMB^hi^ cells compared with CD4^+^GZMB^lo^ cells (Figure 7B). CD4^+^GZMB^+^ CTLs (clusters 2 and 12) also exhibited an upregulated mTORC1 signaling pathway compared with naive-like CD4^+^ T cells (Figure 7B).

Although cell clusters exhibited considerable overlap before and after treatment (Figure 7A), rapamycin led to significant decreases in gene expressions related to cell chemotaxis, inflammatory responses, and the mTORC1 signaling pathway in CD4^+^ CTLs (Figure 7C). Among the 4 cell clusters (clusters 2, 3, 9, and 12) of CD4^+^ CTLs, trajectory analysis showed that after rapamycin treatment, there was enrichment in intermediate differentiated CTLs (cluster 9) and few terminal effectors (cluster 12) (Figure 7D). These results indicated that rapamycin suppressed the mTORC1 pathway, cytotoxic function, and proinflammatory functions in CD4^+^ CTLs while also selectively inhibiting the terminal differentiation of CD4^+^ CTLs. In addition, the GSEA plots of Th1/Th17-polarized cells and T_regs_ exhibited a downregulated trend of mTORC1 signaling pathway after treatment, while no significant difference was shown (Supplemental Figure 6).

## Discussion

In this study, using several in vitro and in vivo models, we have demonstrated that rapamycin suppresses the upregulated mTOR pathways in pathogenic CD4^+^ CTLs and significantly ameliorates GO signs in both mice and patients. Therefore, this “bench to bedside” approach demonstrates the efficacy of the potentially novel agent rapamycin in inhibiting relevant steps in the pathogenesis of GD in general and GO in particular.

Novel targeted therapies are desperately needed for GO (3). As CD4^+^ CTLs were implicated in several immune-mediated fibroinflammatory diseases, such as IgG4-related disease and systematic sclerosis (12, 13, 23, 24), rapamycin is a promising potential intervention for GO, especially to prevent and/or avoid fibrotic complications (25). To our knowledge, this is the first systematic study of rapamycin in GO, and we demonstrate that rapamycin markedly improved GO, which is consistent with previous case reports (26, 27). The rapid and effective suppression of GO-pathogenic CD4^+^ CTLs makes it one of the main mechanisms underlying the remarkable efficacy of rapamycin in GO, although Th1 cells, Th17 cells, and T_regs_ are also reportedly potential targets (28). Moreover, in vitro studies showed that rapamycin can directly suppress fibroblasts and preadipocytes, leading to reductions in fibrosis and adipogenesis, respectively (29, 30). Thus, rapamycin is a plausible therapeutic agent for the management of GO and should be investigated in randomized clinical trials (31).

CD4^+^ CTLs are involved in the process of immune-mediated inflammatory fibrosis. We previously reported that CD4^+^ CTLs expressing GZMB and IFNG can migrate from the circulation to orbital tissues and mediate orbital inflammation and fibrosis (10). Specifically, these CTLs mediate apoptotic death of targeted cells in an HLA class II–restricted manner, followed by overly exuberant tissue repair processes that may lead to fibrosis and tissue dysfunction (23). These CTLs also release proinflammatory cytokines, including IL-6, TNFA, and IL-1B (32–34). These cytotoxic molecules degrade extracellular matrix components, resulting in irreversible tissue remodeling (35, 36).

As we mentioned, the roles of CD4^+^ CTLs have also been described in various inflammatory and fibrotic diseases, i.e., IgG4-related disease, severe COVID-19, and systemic sclerosis. The latter, closely associated with an accumulation of CD4^+^ CTLs in lesions, led to endothelial cell apoptosis and tissue fibrosis (23, 24). Antigen-reactivated CD4^+^ CTLs also infiltrate lesions in fibrosing mediastinitis and drive inflammatory fibrosis (37). The results of these studies illustrated the pathogenicity of CD4^+^ CTLs in the process of immune-mediated inflammatory fibrosis in general and also imply that CD4^+^ CTLs could be potential treatment targets.

While the molecular mechanisms regulating CD4^+^ CTL differentiation are still to be completely elucidated (38, 39), the results of this study support the crucial role of the mTORC1 signaling pathway in the differentiation of effector CD4^+^ CTLs. It is noted that constitutive mTORC1 activation in CD8^+^ T cells results in an increase in terminally differentiated effector cells expressing GZMB and PRF, and rapamycin treatment can inhibit this differentiation (16). In this study, the decrease in the number and the redistributed clusters of CD4^+^ CTLs after rapamycin intervention indicate an important role of mTORC1 in the differentiation of CD4^+^ CTLs.

mTORC1 activity is also required for the production of the proinflammatory cytokines IFNG and TNFA and the expression of killer cell lectin-like receptor G1, which is related to cytotoxicity (16, 40). Proteomic studies revealed the role of mTORC1 in selective expression of effector molecules in CTLs, including granzymes, perforin, TNF, and IFNG (17). Our observation that mTORC1 inhibition by rapamycin suppresses the proinflammatory and cytotoxic functions of CD4^+^ CTLs is consistent with these findings. Thus, mTORC1 pathway is required for CD4^+^ CTLs in both quantity and function.

Beneficial therapeutic effects of rapamycin have been shown in clinical trials of several autoimmune diseases characterized by inflammation and fibrosis. Rapamycin significantly ameliorated dermal fibrosis in diffuse systemic sclerosis, as represented by the modified Rodnan skin thickness score and patient’s global assessment (41). Rapamycin also alleviated nephritis, arthritis, and disease activity in systemic lupus erythematosus (42, 43). In addition, everolimus, a derivative of rapamycin, provided clinical benefit in rheumatoid arthritis (44). The above studies indicate that rapamycin is an effective therapy for alleviating inflammation and fibrosis.

This study had a few limitations. First, the design of the present study did not fully elucidate the mechanism underlying the suppressive effects of rapamycin on CD4^+^ CTLs. Further exploration will be conducted in the future. Second, the clinical study on patients with GO was a case series and not randomized. A larger, multicenter, randomized controlled clinical trial is underway, and efforts to confirm the clinical efficacy and utility of rapamycin are currently ongoing.

In summary, we have shown that rapamycin could decrease the morbidities of GO in mice and improved the clinical conditions of patients with GO. While mTORC1 signaling is the most upregulated mTORC1 signaling in terminally differentiated CD4^+^ CTL effectors, we detected that rapamycin effectively suppressed the number and the proinflammatory and fibrotic functions of these CD4^+^ CTLs. Thus, rapamycin is suggested as a potential immunotherapy for GO and other related autoimmune fibrotic disorders.

## Methods

### Study design.

To study rapamycin as a treatment of GO, we used an established GO mouse model and fed them with rapamycin orally. The effects on the orbits and the thyroid gland were assessed by blood tests and histological and IHC examinations. The frequency and functions of CD4^+^ CTLs were compared using immunofluorescence examinations of the orbital tissues and RNA-Seq of splenocytes. Subsequently, we analyzed the sequencing data of patients with GO in a public database and performed Western blot and flow cytometric analyses of PBMCs from patients with GO. We detected significant upregulation of the mTORC1 pathway in a recently identified, GO-related CD4^+^ CTL population. To determine the effect of the mTORC1 pathway inhibitor rapamycin on GO-specific CD4^+^ CTLs, we cultured PBMCs in vitro and confirmed that rapamycin suppressed CD4^+^ CTL dysfunction using flow cytometry and Western blotting. Finally, we enrolled patients with steroid-refractory GO for low-dose oral rapamycin. Clinical responses and single-cell RNA-Seq data for CD4^+^ PBMCs were investigated to evaluate the potential of rapamycin.

### Mice and viral vectors.

Female BALB/c mice were purchased from Charles River Laboratory. They were housed in cages with filter-top lids and provided with food ad libitum. Mice were maintained at ambient temperature with 12-hour light/12-hour dark cycle (Laboratory Animal Center of Jiaotong University School of Medicine). Adenoviral particles expressing the extracellular fragment of the TSHR (Ad-TSHR289) and Ad-EGFP were reported previously (Baienwei) (11). The gene sequences of TSHR were obtained from the human TSHR289 expression plasmid (psv2-neo-ECE) (UCLA).

### GO mouse model and rapamycin intervention.

Before the start of experiments at the age of 6 weeks, all the mice were allowed to adapt for approximately 1 week and then assigned randomly into 3 groups: the Ad-EGFP, Ad-TSHR A subunit (Ad-TSHRA), and Ad-TSHRA + Rapa groups. Mice in the Ad-TSHRA and Ad-TSHRA + Rapa groups received 10^8^ Ad-TSHR289, while mice in the Ad-EGFP group received Ad-EGFP as a normal control. Each adenovirus was diluted with PBS to 50 μL and injected into the left and right femoral muscles at a volume of 25 mL per injection. Each animal received 9 injections according to a protocol described previously (11).

Rapamycin (DB) was microencapsulated using a spinning disk atomization coating process with the enteric coating material Eudragit S100 (Röhm Pharma), as described previously (45). Encapsulated rapamycin was then incorporated into mouse chow at a concentration of 14 ppm (46). The mice in the Ad-EGFP and Ad-TSHRA groups received a normal diet during the whole experiment. The mice in the Ad-TSHRA + Rapa group received a normal diet at the beginning and then received the diet containing rapamycin from the 11th week to the end of the study.

### Mouse histological, IHC, and immunofluorescence examinations.

After euthanasia, the thyroid gland was removed from the neck in all the mice. Tissue blocks were fixed in 4% neutral paraformaldehyde, dehydrated, and embedded in paraffin. The sections (5 μm) were stained with H&E.

Completely dissected orbital tissue was fixed, decalcified, and then embedded in paraffin. Next, 4 coronal sections with 1 mm intervals across the entire orbit were obtained and then stained with H&E or Masson’s trichrome. The orbital sections were imaged using a 4× objective lens with an Olympus Cue-2 image analysis system connected to an Olympus compound microscope. The areas of fibrosis in the retroocular area were stained blue by Masson’s trichrome. Retroocular adipose tissue could also be seen in H&E-stained sections with the optic nerve as an anatomical landmark. Digitized image analysis of blue pixels was performed using the luminescence tool of Adobe Photoshop software, version CS5. The total fibrosis volume (mm^3^) in the retroocular space of each orbit was calculated, and the area (mm^2^) of retroocular adipose tissue was also calculated by digitized image analysis with the optic nerve as an anatomical landmark.

The primary Abs anti-mouse CD3 (catalog ER80501, Huabio) used for IHC were purchased from Huabio and applied according to the manufacturer’s protocol (Supplemental Table 4). The numbers of CD3^+^ cells in the orbit and thyroid were calculated in 3 random fields at 40× magnification. The primary Abs used for immunofluorescence staining were as follows: anti–mouse/human GZMB (EPR22645-206) (catalog ab255598, Abcam) and anti–mouse/human CD4 (MT310) (catalog sc-19641, Santa Cruz Biotechnology) (Supplemental Table 4). The numbers of CD4^+^GZMB^+^ cells in the orbit and thyroid were also calculated in 3 random fields at 40× magnification.

### Mouse gene expression analysis.

RNA was isolated from the spleen, and differentially expressed genes were identified through fold-change filtering (*P* < 0.05), as described previously (47). To further explore differences, pathway analysis was performed by GSEA using R software (package msigbd, C5). Data related to the RNA-Seq performed using the splenocytes from mice are deposited in the NCBI’s Sequence Read Archive (SRA BioProject PRJNA795042).

### Mouse serological tests.

TRAbs were measured with a commercial kit (Medipan) using the TSH binding inhibition (TBI) assay. TT4 was measured with a commercial kit (Beijing North Institute of Biological Technology) using the competitive RIA method. Measurements were performed according to the manufacturer’s protocol.

### Multicolor immunofluorescence.

Orbital tissues were collected from GO at Tangdu Hospital of Airforce Medical University. Tissue specimens from representative lesions were collected and fixed. Immunofluorescence was performed on 3 μm serial sections of paraffin-embedded tissue after dewaxing, antigen retrieval, and blocking nonspecific binding. The primary Abs used included anti–human/mouse CD4 (MT310) (catalog sc-19641, Santa Cruz Biotechnology), anti-human GZMB (catalog ab4059, Abcam), anti–human/mouse CD90 (catalog GB113753, Servicebio), anti–human/mouse cleaved caspase-3 (Asp175) (5A1E) (catalog 9664, Cell Signaling Technology), and anti–human/mouse HLA-DR (TAL 1B5) (catalog sc-53319, Santa Cruz Biotechnology) (Supplemental Table 4). Secondary Abs were used according to the species origin of the associated primary Ab. Multicolor IHC data were collected on a fluorescence microscope (Nikon Eclipse C1) connected to an imaging system (Nikon DS-U3). Multispectral imaging was performed using CaseViewer at 64× magnification.

### Public data collection and analysis.

Two microarray data sets (GSE9340 and GSE58331) for patients with GO were obtained from the NCBI Gene Expression Omnibus (GEO) and ArrayExpress databases in accordance with our selection criteria on April 1, 2020. GSE9340 was used to analyze thyrocytes from patients with GO (8 GD patients without GO and 10 GD patients with GO). The GSE58331 data set was used to validate the findings for thyrocytes in orbital tissues from patients with GO (28 patients with nonspecific orbital inflammation, 24 patients with GO, and 21 healthy controls).

The GSE9340 and GSE58331 data sets were generated using the GPL6014 and GPL570 platforms, respectively. Normalization and quality control of the data in the data sets were carried out with the limma R package (48). Data analysis was conducted with the limma R package to detect differentially expressed genes between the GD and healthy control data or the thyroid-associated orbitopathy and GD data after normalization. The following cutoff criteria were used: |log_2_ fold-change| > 0.1 and *P* < 0.05. KEGG pathway analysis was conducted with the clusterProfiler R package (49) and a *P* < 0.1 was used as the threshold.

### PBMC collections and rapamycin intervention.

PBMCs were isolated from the peripheral venous blood of patients with newly diagnosed and untreated moderate-to-severe and active GO (CAS ≥ 3). Peripheral venous blood was collected into anticoagulant tubes (BD Biosciences). PBMCs were isolated by Ficoll-Paque (GE Healthcare) density gradient centrifugation. PBMCs were cultured in RPMI 1640 medium (Corning) supplemented with 10% FBS (Gibco) and 1% penicillin-streptomycin-glutamine (Gibco). For in vitro experiments, rapamycin (DB) was dissolved in DMSO and then supplemented with PBMC culture medium at a final concentration of 100 nM. PBMCs were cultured in a cell incubator (37°C, 5% CO_2_) for 48 hours.

### Western blot analysis.

Total protein was extracted using a RIPA lysis buffer kit containing a protease/phosphatase inhibitor cocktail (Tiangen). Protein concentration was measured using the Bicinchoninic Acid (BCA) Assay Kit (Tiangen). Equal amounts of protein were resolved on an SDS-PAGE and then transferred to a PVDF membrane by electrophoretic transfer. The following primary Abs were used: anti-human mTOR (7C10) (catalog 2983, Cell Signaling Technology), anti-human p-mTOR (Ser2448, D9C2) (catalog 5536, Cell Signaling Technology), anti-human p70S6K (49D7) (catalog 2708, Cell Signaling Technology), anti-human p-p70S6K (Thr421/Ser424) (catalog 9204, Cell Signaling Technology), anti-human GAPDH (14C10) (catalog 2118, Cell Signaling Technology), and anti-human β-actin (D6A8) (catalog 8457, Cell Signaling Technology) (Supplemental Table 4). Data were analyzed using ImageJ software (NIH).

### Flow cytometry.

PBMCs from patients were suspended in staining buffer (eBioscience) at a final concentration of 1 × 10^7^ cells/mL. Staining with PE-Cy7–conjugated anti-p-mTOR and PE-conjugated anti–p-S6 Abs was performed as follows: PBMCs were resuspended and fixed in cold 2% paraformaldehyde for 10 minutes at room temperature. The fixed cells were permeabilized and blocked with PBS buffer containing 0.1% Triton X-100 plus 2% BSA for 1 hour at 4°C and then stained with Abs diluted in PBS buffer containing 0.1% Triton X-100 plus 1% BSA. The cells were washed with PBS buffer containing 0.1% Triton X-100 plus 0.5% BSA after each staining step. A cell stimulation cocktail containing a protein transport inhibitor was used for GZMB, PRF1, IFNG, IL-10, TGFB, CX3CR1, and CCL5 staining. All the Abs were used according to the manufacturers’ protocols: FITC anti-human CD4 (catalog 11-0049-08, eBioscience), APC anti-human GZMB (catalog GRB05, eBioscience), PE cy/7 anti-human PRF1 (catalog 25-9994-42, eBioscience), PE cy/7 anti-human IFNG (catalog 25-7319-82, eBioscience), PE anti-human p-S6K (Ser235, Ser236) (catalog 12-9007-41, eBioscience), PE cy/7 anti-human p-mTOR (Ser2448) (catalog 25-9718-41, eBioscience), PE anti-human RORG (catalog 12-6981-80, eBioscience), PE anti-human FOXP3 (catalog 12-4776-42, eBioscience), PerCP cy5.5 anti-human CCL5 (catalog 515507, BioLegend), FITC anti-human T-bet (catalog 644811, BioLegend), PerCP cy5.5 anti-human GATA3 (catalog 653811, BioLegend), PE cy/7 anti-human IL-10 (catalog 501419, BioLegend), PerCP cy5.5 anti-human CX3CR1 (catalog 341614, BioLegend), APC cy/7 anti-human CD4 (catalog 317418, BioLegend), and PE anti-human TGFB1 (catalog 562260, BD Biosciences) (Supplemental Table 4). Flow cytometric analysis was performed using a flow cytometer (FACSAria, BD Biosciences). Data were analyzed by FlowJo software.

### Patients and rapamycin intervention.

The patients with GO with intractable diplopia and not sensitive to combined treatments of intravenous glucocorticoid, radiotherapy, and an oral immunosuppressant (mycophenolic acid or methotrexate) were enrolled (*n* = 5). GO was diagnosed based on clinical presentation and CT examination (3). The severity and activity of disease were assessed using the CAS and European Group on Graves’ Orbitopathy classification (3). Medical records, orthoptic reports, and MRIs were retrospectively reviewed by 2 ophthalmology fellows independently. Each reviewer was unaware of the measurements made by the other. The treating ophthalmologist was involved in resolution when the results assessed by the 2 reviewers were different. The Gorman diplopia scale was used to score the diplopia as following: 1, no diplopia; 2, intermittent gaze-evoked diplopia; 3, gaze-evoked primary-gaze diplopia; and 4, constant primary-gaze (intractable) diplopia. EOMy restriction was scored according to the position of limbus at 9 cardinal gaze photos. We scored 0 for full excursion and −5 for failure to reach the midline (−4 to −1 for excursion in 25% increments), and the scores in the most restricted duction were recorded.

The rapamycin (sirolimus, Pfizer Ireland Pharmaceuticals) was given orally, and concentration in the blood was monitored. The target range of bottom concentration levels was 5–10 ng/mL for patients who received 2 mg daily for 12 months. During the course of rapamycin treatment, the thyroid function of the patients was controlled with an antithyroid drug.

### Single-cell RNA-Seq analysis of patients with GO.

In another 2 refractory patients with GO, the rapamycin was given at 0.5 mg daily for 3 months and the target range of bottom concentration was 2.5–5 ng/mL. PBMCs were suspended in flow cytometry staining buffer (eBioscience) at a final concentration of 10^7^ cells/mL. For cell sorting, PBMCs were prepared as described above. After labeling with anti-CD4 monoclonal Abs (BD Biosciences) for 30 minutes at 4°C in FACS buffer (2% FBS in Dulbecco’s PBS), viable CD4^+^ T cells were sorted into PBS + 0.04% BSA with a FACSAria (BD Biosciences) and retained on ice.

Sorted cells were then resuspended at a concentration of 5 × 10^5^ to 1 × 10^6^ cells/mL with a final viability of greater than 80%. A single-cell library was prepared following the protocol of the v2 reagent kit from 10x Genomics (50), aiming for an estimated 8,000 cells per library. The Cell Ranger software pipeline (version 2.1.1) provided by 10x Genomics was used to demultiplex cellular barcodes, map reads to a genome (GRch38) and transcriptome (STAR aligner), and produce a matrix of gene counts versus cells. We processed the unique molecular identifier count matrix using the R package Seurat (version 4.0.4). After a quality control step, we filtered out 23,317 single cells and 18,474 genes that were included in downstream analyses.

To identify pathogenic CD4^+^ T cell subsets and explore treatment effects on CD4^+^ T cell types, samples collected before (*n* = 2) and after treatment (*n* = 2) were merged, and harmony (version 0.1.0) was used to remove batch effects in each group. The top 4,000 highly variable genes detected by Seurat were selected and further analyzed by principal component analysis to focus on biologically meaningful variations. To visualize the clusters in 2 dimensions, graph-based clustering detection and the UMAP algorithm were applied to the top 8 principal components. The number of components used was determined based on the JackStraw function. The annotations of cell identity for each cluster were defined by the expression of known marker genes. We employed the Monocle (version 2.18.0) algorithm using variable genes of cluster 12 selected by Seurat as the input to determine the differential CD4^+^ CTL states. The CD4^+^ CTL trajectory was inferred using default parameters of Monocle after dimensionality reduction and cell ordering.

### Data and materials availability.

Data related to the RNA-Seq performed using the splenocytes from mice are deposited in NCBI’s SRA accession PRJNA795042. Data related to the single-cell sequencing performed using the CD4^+^ T cells from patients are deposited in NCBI’s SRA accession PRJNA578302.

### Statistics.

Normally distributed data are presented as the mean and SEM intergroup differences were determined by 1-way ANOVA, Mann-Whitney rank-sum tests, and independent-sample 2-tailed *t* tests. Specific statistical methods have been described at the end of the figure legends. All statistical analyses were performed using SPSS for Windows (SPSS 19.0), and a *P* value less than 0.05 was considered significant.

### Study approval.

This study followed the Declaration of Helsinki and ethical approval was obtained from the institutional review board. All procedures performed in studies involving patients and healthy volunteers were in accordance with the ethical standards of the First Affiliated Hospital of Xi’an Jiaotong University (XJTU1AF2016LSK-35, XJTU1AF2017LSK-44) and the Chinese University of Hong Kong (KC/KE-10-0218/ER-3, CRE-2010.594). All the human participants provided written informed consent.

All animal experiments were approved by the local animal welfare authority and Ethics committee of the Medical Department of Xi’an Jiaotong University (IACUC 2015036). All the animal care and treatments were in accordance with the *Guide for Care and Use of Laboratory Animals* (National Academies Press, 2011).

## Author contributions

YW, BYS, and KY acquired the funds. YW and MZ designed the experiments. MZ and ZYC performed the in vitro experiments and animal experiments. KKHL and KKLC recruited the patients. ZYC, YFL, YYK, and KY analyzed the sequencing data. HG, YJL, TTS, and MQH provided the GO patient samples. MZ and ZYC wrote the original manuscript. YW, BYS, KKLC, GJK, and KY reviewed and edited the manuscript. All the 3 co–first authors contributed significantly to the study, and the order was based on their contributions: MZ is designated first coauthor as she performed all long-term in vitro experiments, performed animal experiments, wrote the original manuscript, and made the revisions; KKLC is designated second coauthor as he performed the recruitment of all long-term patients, intervention, and follow-up; ZYC is designated third coauthor as she performed the in vitro experiments, analyzed sequencing data, and wrote the original manuscript. All authors participated in data analysis and preparation of the manuscript.

## Supplementary Material

Supplemental data

## Version 1 12/29/2022

In-Press Preview

## Version 2 02/08/2023

Electronic publication
