# Supplementary material for: Rapamycin improves Graves’ orbitopathy by suppressing CD4+ cytotoxic T lymphocytes
Source: JCI Insight. 2023 Feb 8;8(3):e160377. doi: 10.1172/jci.insight.160377 (PMC9977423; doi:10.1172/jci.insight.160377)
Supplement: Supplemental data [file jciinsight-8-160377-s023.pdf]

Supplementary table 1 Comparisons of gene expression between different groups.

Type	Genes	*P*-adj [a] (control group vs. GO model group)	*P*-adj (control group vs. rapamycin treatment group)
Immune Response	THEMIS	0.003247	0.145851
	TRAT1	0.017754	0.082263
	ITK	0.002208	0.100777
	TXK	0.013620	0.096480
Cell-Cell Adhesion	LEF1	0.008076	0.129240
	CD247	0.003968	0.232243
	TNR	0.010939	0.271147
Inflammation and Chemotaxis	IFNG	0.022515	0.212678
	CCL5	0.015533	0.193604
	CCR7	0.046903	0.053325
	CXCR6	0.001652	0.209455
	CXCR3	0.003570	0.588464
	CXCL10	0.032633	0.166478
Cytolysis and Cell Killing	GZMM	0.005989	0.415911
	GZMK	0.006840	0.151235
	PRF1	0.036242	0.156871
	ALB	0.001302	0.180068
	F2RL1	0.012858	0.119809

Expressed gene analysis was performed for mouse splenocytes. Compared with the Ad-EGFP group, the GO model group showed significantly increased gene expression related to the immune response, cell-cell adhesion, inflammation, chemotaxis, cytolysis and cell killing ($p<0.05$), while the rapamycin treatment group showed no significant differences ($p>0.05$).

[a] *P*-adj: adjusted *P* value.

Supplementary table 2. The orbital pathology in GO model and rapamycin treatment groups.

Groups	Fibrosis [a] (mm^3)	Adipogenesis [b] (mm^2)	Orbitopathy [c]
GO model	0.3186	**0.06088**	+
GO model	**0.4088**	**0.04994**	+
GO model	0.3249	0.03885	−
GO model	**0.4552**	**0.05946**	+
GO model	**0.4195**	**0.06884**	+
GO model	0.3253	**0.05042**	+
GO model	**0.4086**	**0.04710**	+
GO model	**0.5069**	0.03372	+
Rapamycin treatment	0.2690	0.04187	−
Rapamycin treatment	0.2393	0.04089	−
Rapamycin treatment	**0.4753**	**0.04788**	+
Rapamycin treatment	0.2473	0.02733	−
Rapamycin treatment	0.3034	**0.05153**	+
Rapamycin treatment	0.2398	0.03152	−
Rapamycin treatment	**0.4763**	0.03569	+
Rapamycin treatment	0.2629	0.03135	−

[a] Fibrosis: control mice mean+2 S.D.=0.3352 mm^3. higher values (bold) indicated significant positivity.

[b] Adipogenesis: control mice mean+2 S.D.=0.04553 mm^2. higher values (bold) indicated significant positivity.

[c] Orbitopathy: +: existing orbital pathogenesis including fibrosis and adipogenesis. −: no evidence of fibrosis and adipogenesis.

Supplementary table 3. Clinical information of steroid-refractory GO patients treated by rapamycin.

No.	Age/ Sex [a]	Thyroid condition [b]	Orbitopathy onset (months) [c]	Tough levels (ug/l) [d]		CAS [e]	Diplopia [f]	EOMy [g]
1	58/M	EGO	11	3.9	Pre	1	4	-3
					Post	0	4	-3
2	45/M	GO	16	N/A	Pre	1	4	-3
					Post	0	3	-1
3	43/M	GO	10	5.3	Pre	2	4	-2
					Post	0	1	-1
4	33/F	GO	11	6.5	Pre	4	4	-1
					Post	0	2	0
5	57/F	GO	28	7.1	Pre	1	4	-3
					Post	0	4	-3

[a] Age/Sex: Age: Age of disease onset. M: Male. F: Female.

[b] Thyroid condition: EGO=Euthyroid Graves orbitopathy, GD=Grave's disease.

[c] Orbitopathy onset: the time between orbitopathy onset and the use of rapamycin.

[d] Tough levels: the aimed tough levels of rapamycin were 5-10 ng/ml for patients who received 2 mg daily for 12 months.

[e] CAS: Clinical activity score.

[f] Gorman diplopia scale was used to score the diplopia as following: 1, no diplopia; 2, gaze - evoked diplopia; 3, intermittent primary-gaze diplopia; 4, constant primary-gaze (intractable) diplopia.

[g] EOMy restriction was scored according to the position of limbus at 9 cardinal gaze photos. We scored 0 for full excursion and −5 for failure to reach the midline (−4 to −1 for excursion in 25% increments), and the scores in the most restricted duction were recorded.

Supplementary table 4. Antibodies used in the methods section.

	antibody	catalog#	Company
Western blot	anti-human mTOR (7C10)	2983	CST [a]
	anti-human p-mTOR (Ser2448, D9C2)	5536	CST
	anti-human p70S6K (49D7)	2708	CST
	anti-human p-p70S6K (Thr421/Ser424)	9204	CST
	anti-human GAPDH (14C10)	2118	CST
	anti-human β-actin (D6A8)	8457	CST
Flow cytometry	FITC anti-human CD4	11-0049-08	eBioscience
	APC anti-human GZMB	GRB05	eBioscience
	PE cy/7 anti-human PRF1	25-9994-42	eBioscience
	PE cy/7 anti-human IFNG	25-7319-82	eBioscience
	PE anti-human p-S6K (Ser235, Ser236)	12-9007-41	eBioscience
	PE cy/7 anti-human p-mTOR (ser 2448)	25-9718-41	eBioscience
	PE anti-human RORG	12-6981-80	eBioscience
	PE anti-human FOXP3	12-4776-42	eBioscience
	PerCP cy5.5 anti-human CCL5	515507	Biolegend
	FITC anti-human Tbet	644811	Biolegend
	PerCP cy5.5 anti-human GATA3	653811	Biolegend
	PE cy/7 anti-human IL10	501419	Biolegend
	PerCP cy5.5 anti-human CX3CR1	341614	Biolegend
	APC cy/7 anti-human CD4	317418	Biolegend
	PE anti-human TGFB1	562260	BD
Histological examination	anti-mouse CD3	ER80501	Huabio
	anti-human/mouse CD4 (MT310)	sc-19641	Santa Cruz
	anti-human/mouse HLA-DR (TAL 1B5)	sc-53319	Santa Cruz
	anti-human/mouse GZMB (EPR22645-206)	ab255598	Abcam
	anti-human GZMB	ab4059	Abcam

	anti-human/mouse cleaved caspase-3 (Asp175) (5A1E)	9664	CST
	anti-human/mouse CD90	GB113753	Servicebio

[a] CST: Cell Signaling Technology

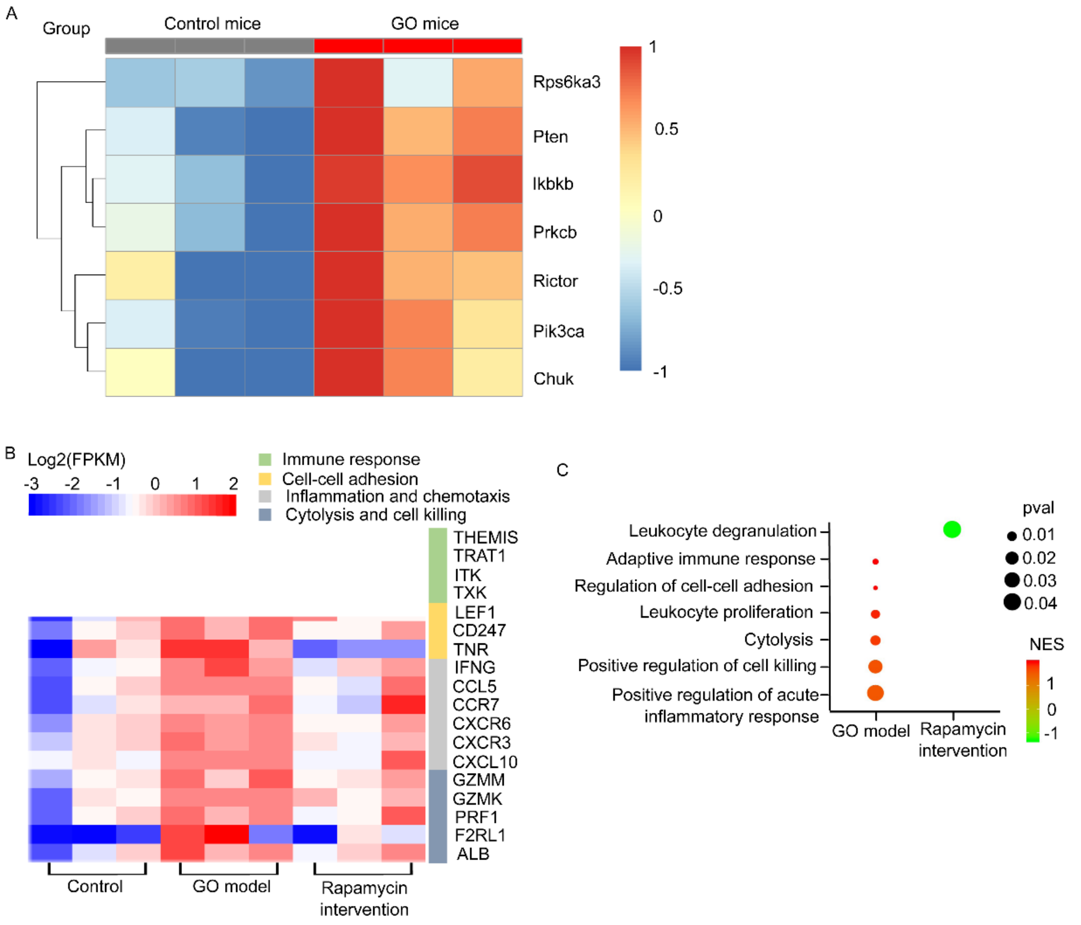

Supplementary Figure 1: RNA-sequencing results of splenocytes of mice (n=3 for each group). (**A**) Heatmap of gene expression related to mTOR signaling pathway in splenocytes from mice. Adjusted $P < 0.05$. (**B**) Heatmap of gene expression related to the immune response, cell-cell adhesion, inflammation, chemotaxis, cytolysis and cell killing in splenocytes from the three groups of mice. The fragments per kilobase per million (FPKM) of expressed genes were indicated by color. (**C**) Gene set enrichment analysis (GSEA) for the indicated pathways in the transcriptome of GO model and rapamycin intervention mice versus control mice. P values indicated by circle size. Normalized enrichment scores (NES) of indicated pathways were indicated by color, and the positive scores indicated positive correlation.

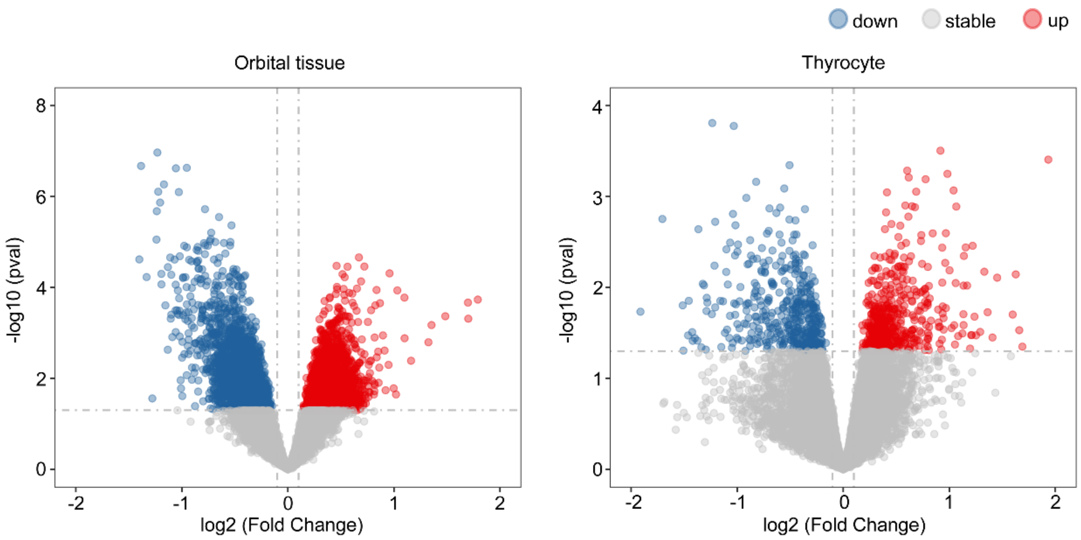

Supplementary Figure 2: Volcano plots of DEGs in the orbital tissues and thyrocytes of GO patients. Two microarray datasets (GSE9340 and GSE58331) of GO patients were obtained from the NCBI Gene Expression Omnibus (GEO) and ArrayExpress and analyzed. There were 4,861 and 690 differentially expressed genes (DEGs) in the GO gene set identified in the GSE58331 dataset (orbital tissues: GO vs. HC) and in the GSE9340 dataset (thyrocytes: GO vs. GD), respectively.

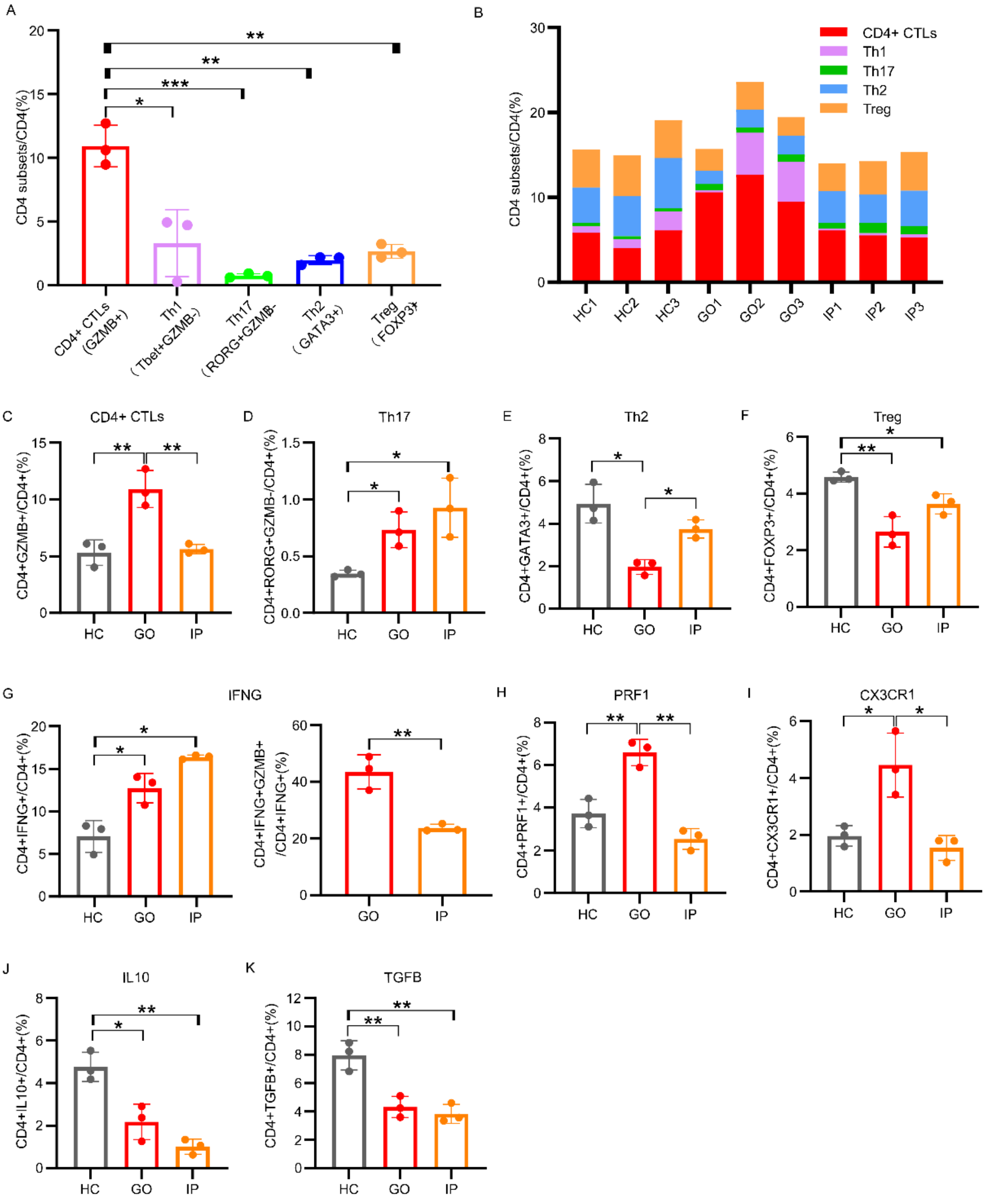

Supplementary Figure 3: The proportion of CD4+ CTLs was increased in the PBMCs of GO patients. (A) Flow cytometric analysis of the proportions of major CD4+ T cells subsets in the PBMCs of GO patients**. (B-F)** Flow cytometric analysis of the proportions of major CD4+ T cells subsets in the PBMCs of healthy controls (HC), GO patients and orbital inflammatory pseudotumor (IP) patients (n=3)**.** (**G-I**) Flow cytometric analysis of the proportions of CD4+IFNG+, CD4+PRF1+ and CD4+CX3CR1+ cells in the PBMCs of HC, GO patients and IP patients (n=3); these phenotypes indicated the cell functions of inflammation, cytotoxicity and chemotaxis, respectively. (**J-K**) Flow cytometric analysis of the proportions of CD4+IL10+ and CD4+TGFB+ cells in the PBMCs of HC, GO patients and IP patients (n=3); these phenotypes indicated the cell functions of Tregs. Values represent the mean±SEM. Two-tailed, independent samples t-tests (**A-K**): *P<0.05, **P<0.01, ***P<0.001.

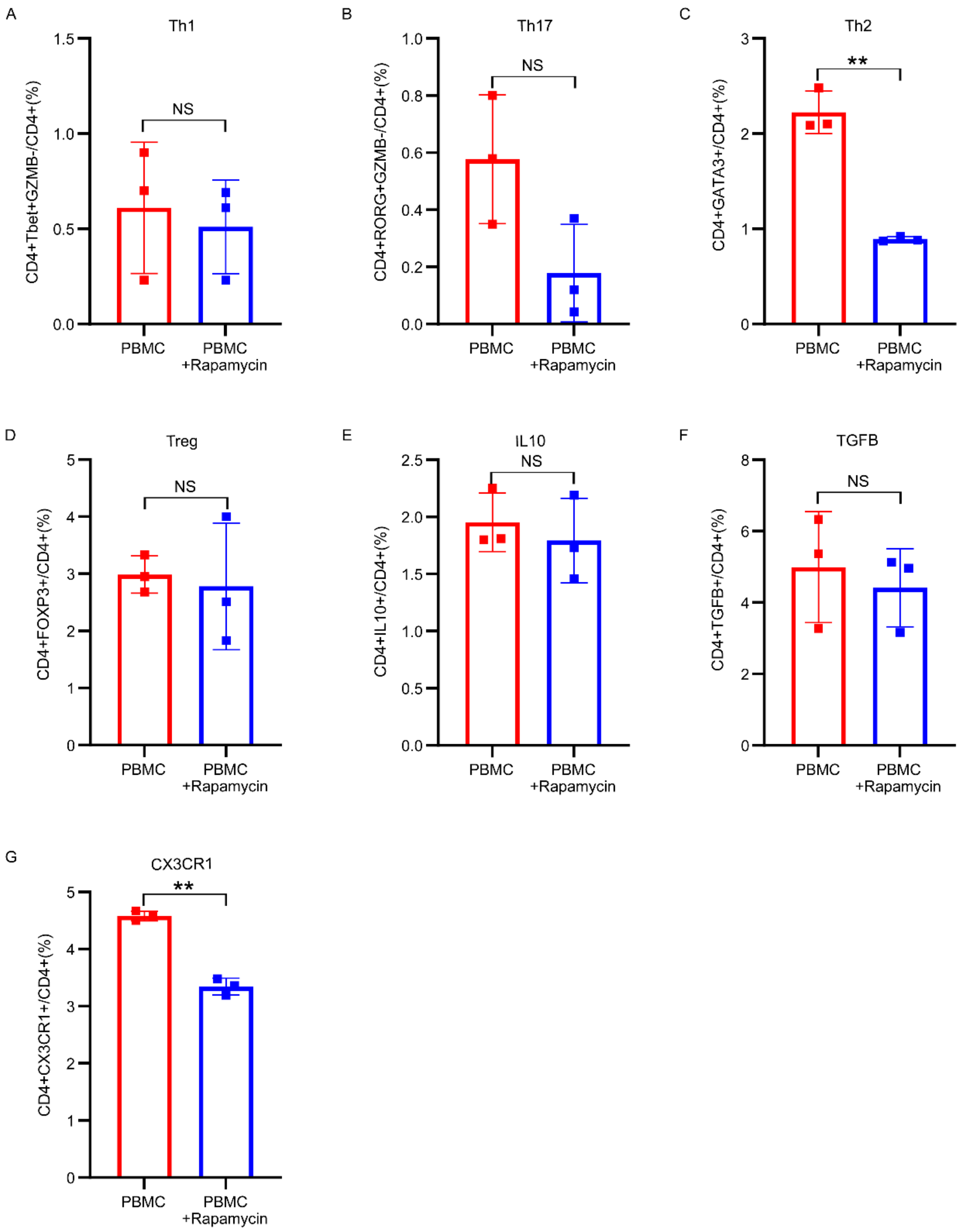

Supplementary Figure 4: Flow cytometric analysis of the proportions and functions of other major CD4+ T cell subsets in PBMC after the treatment of rapamycin. (A-C) Flow cytometric analysis of the proportions of Th1、Th2 and Th17 in the PBMCs after the treatment of rapamycin (n=3). **(D-F)** Flow cytometric analysis of the proportions of Tregs, CD4+IL10+ and CD4+TGFB+ cells in the PBMCs after the treatment of rapamycin (n=3)**.** (**G**) Flow cytometric analysis of the proportions of CD4+CX3CR1+ cells in the PBMCs after the treatmentof rapamycin (n=3). Values represent the mean±SEM. Two-tailed, independent samples t-tests (**A-G**): ***P*<0.01.

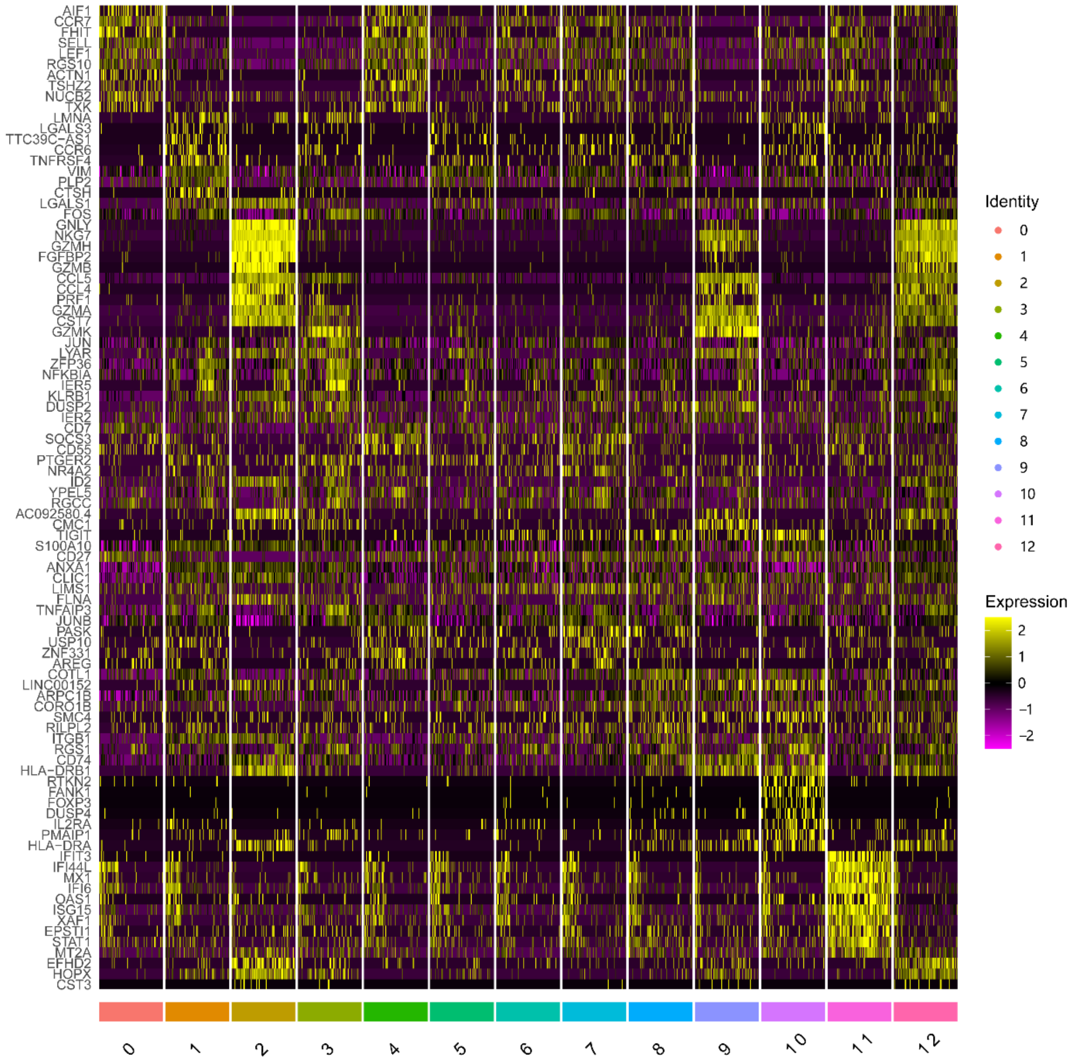

Supplementary Figure 5：Heatmap of the top 20 genes per cluster with the number of DEGs indicated for the GO patients. Adjusted $P < 0.05$.

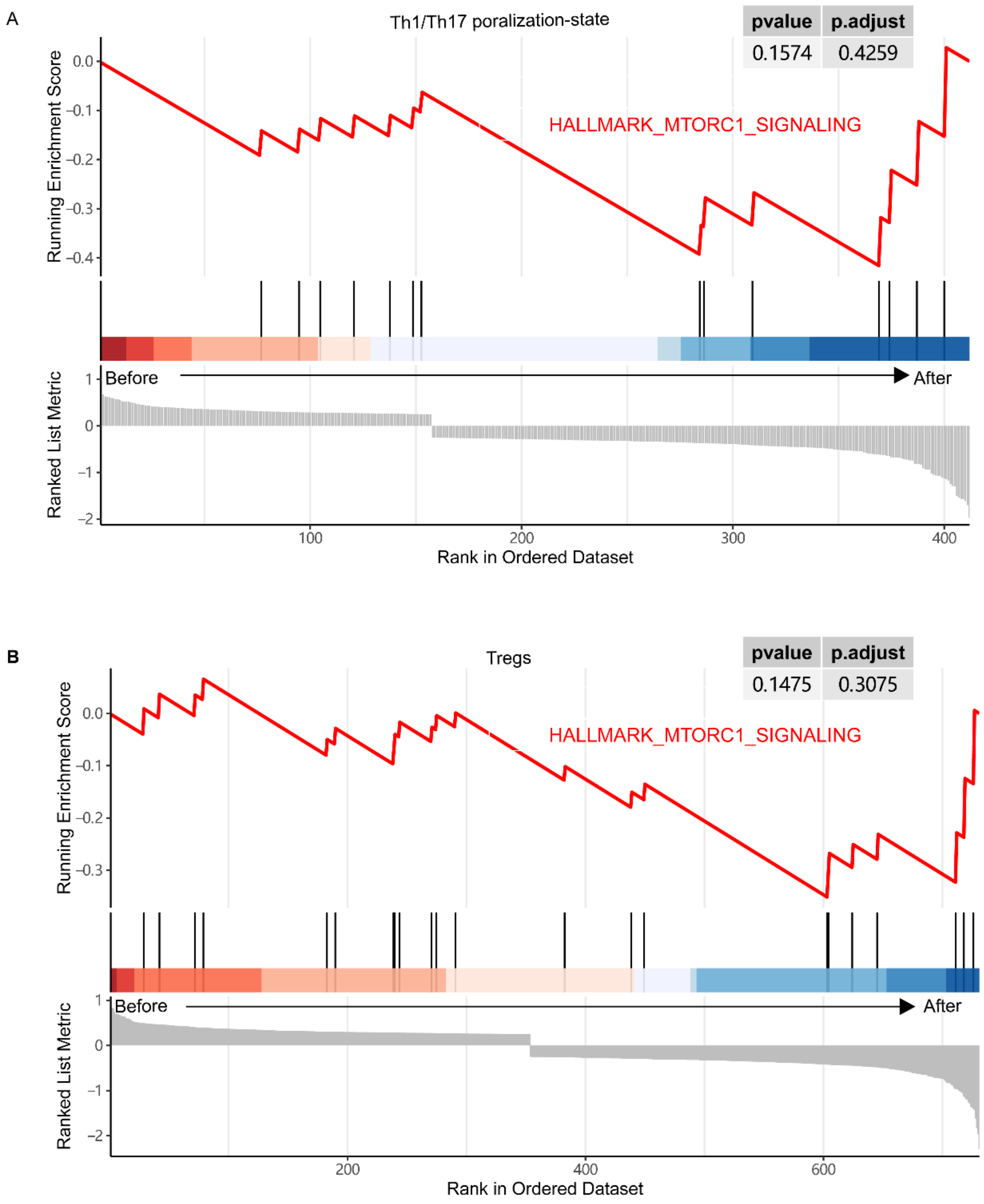

Supplementary Figure 6：GSEA enrichment plots for mTORC1 signaling gene set in the transcriptome of Th1/Th17-polarized cells and Tregs. (**A**) GSEA enrichment plots for mTORC1 signaling gene set in the transcriptome of Th1/Th17-polarized cells before and after treatment. （**B**） GSEA enrichment plots for mTORC1 signaling gene set in the transcriptome of Tregs before and after treatment.
